# Cook County Hospital

**Published:** 1886-04

**Authors:** 


Cook County Hospital.—An adjourned meeting of the Board of County Commissioners was held on Saturday, February 27, Commissioner Klehm in the chair.

The Committee onHospitals asked the appointment of the following physicians upon the medical staff of the County Hospital, to constitute the Medical Board of that institution:

Surgeons—Christian Fenger, J. B. Murphy, A. J. Baxter, J. M. Hutchinson, W. P. Verity, Leonard St. John, D. A. K. Steele, E. W. Lee, Truman W. Miller.

Physicians—Norman Bridge, P. J. Rowan, H. C. Kerber, A. C. Cotton, S. A. McWilliams, G. B. Abbott, John A. Robison, D. W. Nolan, Andrew J. Coey.

Obstetricians and Gynæcologists—John Guerin, Ferd Henrotin, F. C. Schaefer George H. Randell.

Ophthalmologist and Otologist—W. F. Smith.

Pathologist—Elbert Wing.

The report was adopted.

